# Impact of Rehabilitation on Breast Cancer Related Fatigue: A Pilot Study

**DOI:** 10.3389/fonc.2020.556718

**Published:** 2020-10-21

**Authors:** Marco Invernizzi, Alessandro de Sire, Lorenzo Lippi, Konstantinos Venetis, Elham Sajjadi, Francesca Gimigliano, Alessandra Gennari, Carmen Criscitiello, Carlo Cisari, Nicola Fusco

**Affiliations:** ^1^Physical and Rehabilitative Medicine, Department of Health Sciences, University of Eastern Piedmont, Novara, Italy; ^2^Rehabilitation Unit, “Mons. L. Novarese” Hospital, Vercelli, Italy; ^3^Division of Pathology, IEO, European Institute of Oncology IRCCS, Milan, Italy; ^4^Ph.D. Program in Translational Medicine, University of Milan, Milan, Italy; ^5^Department of Mental and Physical Health and Preventive Medicine, University of Campania ‘Luigi Vanvitelli', Napoli, Italy; ^6^Division of Medical Oncology, University Hospital “Maggiore della Carità”, Novara, Italy; ^7^New Drugs and Early Drug Development for Innovative Therapies Division, IEO, European Institute of Oncology IRCCS, Milan, Italy; ^8^Physical Medicine and Rehabilitation Unit, University Hospital “Maggiore della Carità”, Novara, Italy; ^9^Department of Oncology and Hemato-Oncology, University of Milan, Milan, Italy

**Keywords:** breast cancer, quality of life, rehabilitation, fatigue, muscle strength, muscle performance, precision medicine

## Abstract

Breast cancer fatigue (BCF) is a complex and multidimensional condition characterized by a persistent sense of physical and/or mental stiffness, resulting in a substantial impairment of health-related quality of life in breast cancer survivors. Aim of this prospective cohort study was to evaluate the feasibility and the effectiveness of a 4-week rehabilitation protocol on BCF, muscle mass, strength, physical performance, and quality of life in breast cancer (BC) survivors. We recruited adult BC women with a diagnosis of BCF, according to the International Classification of Diseases 10 criteria, referred to the Outpatient Service for Oncological Rehabilitation of a University Hospital. All participants performed a specific physical exercise rehabilitative protocol consisting of 60-min sessions repeated 2 times/week for 4 weeks. All outcomes were evaluated at the baseline (T0), at the end of the 4-week rehabilitation treatment (T1), and at 2 months follow up (T2). The primary outcome measure was the Brief Fatigue Inventory (BFI); secondary outcomes included: Fat-Free Mass and Fat Mass, assessed by Bioelectrical Impedance Analysis (BIA); Hand Grip Strength Test (HGS); Short Physical Performance Battery (SPPB); 10-meter walking test (10 MWT); 6-min walking test (6 MWT); European Organization for Research and Treatment of Cancer Quality of Life Questionnaire (EORTC QLQ–C30). Thirty-six women (mean age: 55.17 ± 7.76 years) were enrolled in the study. Significant reduction of BCF was observed both after the 4-week rehabilitation treatment (T1) (BFI: 5.4 ± 1.6 vs. 4.2 ± 1.7; *p* = 0.004) and at the follow-up visit (T2) (BFI: 5.4 ± 1.6 vs. 4.4 ± 1.6; *p* = 0.004). Moreover, significant differences (*p* < 0.001) HGS, SPPB, 10 MWT, 6 MWT, and EORTC QLQ-C30 were found at T1, while at T2 all the outcome measures were significantly different (*p* < 0.05) from the baseline. The rehabilitation protocol seemed to be feasible, safe, and effective in reducing BCF, improving muscle mass and function, and improving HRQoL in a cohort of BC survivors. The results of this study could improve awareness of this underestimated disease, suggesting the definition of a specific therapeutic exercise protocol to reduce BCF.

## Introduction

Breast cancer is the most common cancer in women and one of the leading causes of cancer-related death worldwide (1). Owing to the advances in the clinical management of this tumor, the number of long-term survivors has progressively increased during the past four decades (1). In this scenario, health-related quality of life has become more and more important in the overall patients' outcome evaluation (2–6).

Cancer-related fatigue, also known as cancer fatigue, is a highly prevalent long-term side effect among breast cancer survivors (7, 8). This complex and multidimensional condition is clinically characterized by a persistent sense of physical, emotional, and/or cognitive stiffness, resulting in a substantial impairment of health-related quality of life (7, 9). The etiology of breast cancer fatigue (BCF) is poorly understood and probably related to mitochondrial dysfunction, inflammation, and increased reactive oxygen species production (8, 10, 11). However, the wide subjectivity of BCF hinders further research to explain its pathogenesis. Several risk factors have been identified so far, including low socioeconomic status, sleep disturbance, emotional stress, anxiety, physical inactivity, high body mass index (BMI), radical surgery, chemotherapy, and radiotherapy (8, 9). According to the American Society of Clinical Oncology (ASCO) and the National Comprehensive Cancer Network (NCCN) guidelines, specific screening programs for BCF should be performed (7, 12). In this respect, the gold standard for the evaluation of BCF is self-reporting using scales, questionnaires, and/or inventories (13). Regrettably, the great heterogeneity in these diagnostic methods, coupled with the lack of widely adopted guidelines, represents a major limitation in the clinical management of BCF (13, 14).

Several types of interventions have been proposed to treat or reduce BCF, including counseling, psycho-education, physical and mind-body activity, massage therapy, acupuncture, music therapy, supplements (e.g., ginseng, vitamin D, psychostimulants), and physical exercise (13). Among these, the supervised physical exercise is supported by the strongest evidence of safety and effectiveness in reducing BCF (15–19). Nevertheless, the optimal exercise interventions scheme (i.e., type, combination, frequency, intensity, and duration) to reduce BCF remains controversial. The aim of this study was to evaluate the feasibility and effectiveness of a 4-week rehabilitation protocol on BCF reduction.

## Materials and Methods

### Patients

This prospective cohort study involved a consecutive series of breast cancer survivors suffering from BCF. All patients referred to the Outpatient Service for the Oncological Rehabilitation of the Physical Medicine and Rehabilitation Unit of University Hospital “Maggiore della Carità” in Novara, Italy over a 24-month period, from January 2018 to December 2019. The inclusion criteria were the following: (1) diagnosis of invasive breast cancer (2) surgery performed at least 12 months earlier; (3) diagnosis of cancer fatigue according to the International Classification of Diseases Tenth Revision (ICD-10) criteria. The exclusion criteria were the following: (1) anemia, defined as hemoglobin <9 g/dl; (2) severe thrombocytopenia, defined as platelets <100,000/mm^3^; (3) history of bleeding; (4) hypothyroidism without replacement therapy; (5) persistent insomnia; (6) central nervous system primary and/or metastatic tumors. Inclusion and exclusion criteria are summarized in Table 1. The study protocol was approved by the local Institutional Review Board and was compliant with the ethical guidelines of the responsible governmental agency. At the enrollment, all the participants were asked to carefully read and sign an informed written consent. The investigators provided to protect the privacy and the study procedures according to the Declaration of Helsinki.

**Table 1:** Eligibility criteria of the study population.

**Inclusion criteria**	
(1) Diagnosis of invasive breast cancer	Patients with a diagnosis of breast cancer with cancer cells that have grown through the lining of the ducts into the surrounding breast tissue.
(2) Surgery at least 12 months earlier	All patients underwent breast surgery (conservative or mastectomy) at least 12 months earlier.
(3) Diagnosis of cancer fatigue according to the International Classification of Diseases Tenth Revision (ICD-10) criteria	The ICD-10 criteria define cancer-related fatigue (CRF) as diminished energy, an increasing need for rest, limb heaviness, diminished ability to concentrate decreased interest in engaging in normal activities, sleep disorder, inertia, emotional liability, perceived problems with short-term memory, and post-exertional malaise exceeding several hours.
**Exclusion criteria**	
(1) Severe anemia	A severe decrease in hemoglobin blood levels defined by the threshold of <9 g/dl.
(2) Severe thrombocytopenia	A severe decrease of thrombocyte blood levels defined by the threshold of <100,000/mm^3^.
(3) History of bleeding	Patients that have a history of bleeding during cancer evolution.
(4) Hypothyroidism without replacement therapy	An endocrine system disorders, where the thyroid produces insufficient levels of thyroid hormone, leading to several symptoms, including fatigue.
(5) Persistent insomnia	Insomnia lasting more than 1 month, that might result in increased fatigue.
(6) Central nervous system primary and/or metastatic tumors	Other tumors that might affect patients.

### Intervention

**Figure 1:**
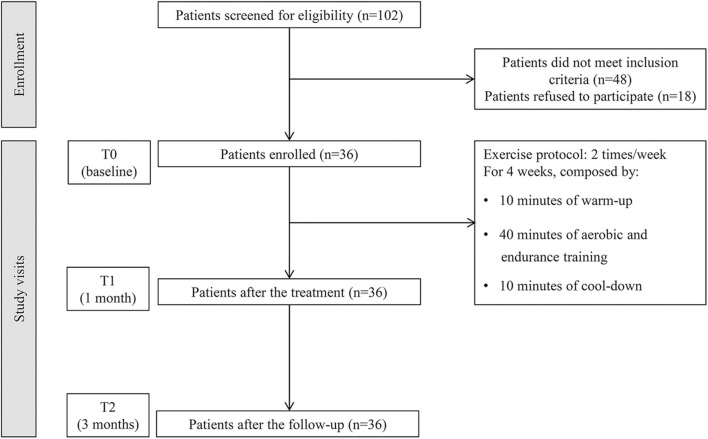
Study flow chart.

All participants were subjected to a specific physical exercise rehabilitative protocol consisting of 10 min of warm-up, 40 min of aerobic exercise (e.g., walking, cycling, rowing) and strength training (e.g., light weightlifting), and 10 min of cool-down. Each session was repeated 2 times/week with at least 2 days of rest for 4 weeks, under the supervision of an experienced physical therapist. The study flow chart is shown in Figure 1. At the end of the rehabilitation treatment, a booklet encompassing the pictures and instructions of the previously performed exercises was provided to the patients. To maintain the benefits obtained during the hospital treatment, all patients were trained and strongly encouraged to continue the exercises at home. In the case of BCF evolution and/or worsening of general clinical conditions, the rehabilitation treatment was stopped.

### Fatigue and Physical Performance Evaluation

At the baseline (T0), demographic and anthropometric characteristics, cancer location and staging, as well as pharmacologic history, have been assessed. All outcomes were also evaluated at the end of the 4-week rehabilitation treatment (T1), and at 2 months follow-up (T2).

#### Primary Outcome

The primary outcome measure was the Brief Fatigue Inventory (BFI), a multidimensional self-report scale that assesses the effects of fatigue on health-related quality of life originally reported by Mendoza et al. (20, 21). This survey is composed of nine questions scored on a 0–10 point scale. The BFI is presented as two parts. Specifically, the first three questions rate the current, usual, and worst levels of fatigue over the last 24 h, while the remaining six questions are related to the impact of fatigue on activity, mood, walking, work, relationships, and enjoyment of life. A total BFI score is then calculated by the mean of the nine scores, where scores 1–3 indicate slight fatigue, scores 4–6 moderate fatigue, and scores 7–10 severe fatigue.

#### Secondary Outcomes

The secondary outcomes were the following. (1) Body composition in terms of fat-free mass (FFM) and fat mass (FM) by bioelectrical impedance analysis (BIA). For this study, the BIA101 Anniversary (Akern Srl, Pontassieve, Florence, Italy) was used. BIA evaluations were performed with patients in a supine position, with the upper and lower limbs abducted by about 30 and 45 degrees, respectively. The electrodes were placed on hands and feet at a minimum distance of 5 cm and connected to the cable with the red insulated tweezers (distal) and black (proximal). FFM and FM were determined according to the equation elaborated by Kyle et al. (22). (2) Handgrip strength test (HGS), using the Jamar® hydraulic hand dynamometer (Sammons Preston, Rolyon, Bolingbrook, IL, USA) to assess the isometric grip strength of the hand, according to the American College of Sports Medicine recommendations (23). This measure strongly correlates with global muscle strength (24). Briefly, the test was conducted with the participant seated on a chair, the shoulder adducted and neutral for rotation, with the elbow flexed at 90°, the forearm neutral for prono-supination and wrist extension between 0 and 30° with 0–15 degrees of ulnar deviation. The test was repeated three times to obtain the mean. (3) Short physical performance battery (SPPB), a composite scale ranging from 0 to 12, assessing walking speed, standing balance, and sit-to-stand performance (25, 26). (4) Ten-meter walking test (10MWT) to assess walking speed (27). (5) 6-min walking test (6 MWT) for the integrated response of cardiopulmonary and musculoskeletal systems (28). (6) European Organization for Research and Treatment of Cancer 30-item quality of life questionnaire (EORTC QLQ–C30), a unidimensional scale that assesses the severity of symptoms related to cancer and its treatment, consisting of functional scales (i.e., physical, role, cognitive, emotional, and social functioning), a global quality of life scale, symptom scales (i.e., fatigue, nausea and vomiting, and pain), and global health (i.e., appetite loss, diarrhea, dyspnea, constipation, insomnia, financial impact) (29). Furthermore, at T1, both the enrolled patients and the physical therapist expressed their satisfaction regarding this treatment, which was assessed using the Global perceived effect (GPE) Scale, ranging from 1 (best satisfaction) to 7 (unsatisfaction) (30).

### Statistical Analysis

Statistical analyses were performed using GraphPad Prism®, version 7.00 (GraphPad Software, La Jolla California USA). Due to the low numerosity of the sample, we assumed a non-gaussian distribution of the considered variables, as previously described (31). Differences between single variables at different time-points were assessed by the two-way Friedman Analysis of Variance (ANOVA) for repeated measure and Dunn's *post hoc* test. A type I error level of 0.05 was chosen. A *p*-value lower than 0.05 was considered statistically significant.

## Results

A total of 102 women with BCF were assessed. Among them, 48 (47%) did not meet the eligibility criteria and 18 (18%) refused to sign the informed consent. Taken together, 36 patients were enrolled in the study. The study flowchart is depicted in Figure 1.

### Demographic and General Characteristics of BCF Patients

The mean age at diagnosis of the 36 patients included in this study was 55.17 ± 7.76 years. Most of the women were of normal weight or borderline overweight (BMI = 25.15 ± 5.52 kg/m^2^). The rate of smokers was similar to that of the general women population (*n* = 8, 22.2%). All of them underwent breast surgery, with equal distribution between conservative and radical surgery (*n* = 19, 52.7%, and *n* = 17, 47.3%, respectively). The en bloc axillary dissection was performed in 16 (44.4%) patients, while 21 (58.3%) were subjected to radiotherapy, either in the supraclavicular fossa or in the chest wall. Breast cancer related lymphedema was present in 12 (33.3) patients. The baseline characteristics along with the therapeutic information are listed in Table 2.

**Table 2:** Clinicopathologic and demographic characteristics of the patients included in this study.

	**Patients (*n* = 36)**
Age	55.17 ± 7.76 years
Body mass index (BMI)	25.15 ± 5.52 kg/m^2^
Smoke (*n*, %)	8 (22.2)
**Breast surgery**	
Conservative (*n*, %)	19 (52.7)
Mastectomy (*n*, %)	17 (47.3)
**Axillary surgery**	
Sentinel lymph node (n, %)	20 (55.6)
*En bloc* dissection (n, %)	16 (44.4)
Radiotherapy (*n*, %)	21 (58.3)
Chemotherapy (*n*, %)	26 (72.2)
Hormone therapy (*n*, %)	29 (80.6)
Trastuzumab (*n*, %)	10 (27.7)
Upper limb lymphedema (*n*, %)	12 (33.3)

### Reduction of BCF After Rehabilitation Treatment

**Figure 2:**
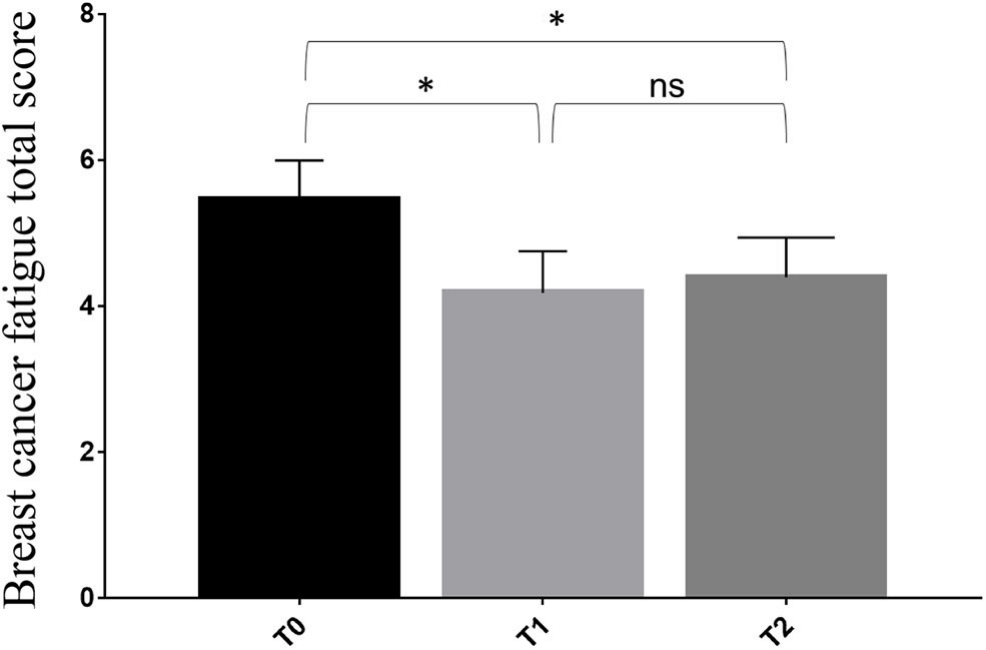
Differences in primary outcome measure from the baseline (T0) to the end of 4-week rehabilitation treatment (T1) and the follow-up assessment at 3 months from the baseline (T2). * *p* < 0.05; ns, non significant.

We observed a statistically significant reduction of the BCF score after the 4-week rehabilitation treatment (T1) compared to T0 (4.2 ± 1.7 vs. 5.4 ± 1.6; *p* = 0.004). Despite the small sample size, the significance was substantially maintained at the follow-up visit (T2) (4.4 ± 1.6; *p* = 0.004), as showed in Figure 2 and Table 3. However, no statistical significance was observed between the T1 and theT2 stage. Furthermore, we found significant differences at T1 in terms of HGS (20.1 ± 5.8 vs. 22.5 ± 5.2: *p* < 0.001), SPPB (9.3 ± 2.0 vs. 11.3 ± 1.2; *p* < 0.001), 10 MWT (1.5 ± 0.3 vs. 1.8 ± 0.3; *p* < 0.001), 6 MWT (464.5 ± 62.9 vs. 554.1 ± 71.6; *p* < 0.001), EORTC QLQ-C30 Functional score (69.2 ± 14.9 vs. 76.9 ± 15.7; *p* < 0.001), EORTC QLQ-C30 Symptoms score (29.2 ± 14.9 vs. 21.2 ± 16.0: *p* < 0.001), and EORTC QLQ-C30 Global Health score (40.7 ± 12.5 vs. 67.6 ± 14.8; *p* < 0.001). At 2 months (T2), all the outcome measures significantly differ from the baseline (*p* < 0.05), including FFM (43.2 ± 6.4 vs. 45.5 ± 6.6; *p* < 0.001) and FM (24.0 ± 10.6 vs. 21.7 ± 10.0; *p* < 0.001), as showed by Table 3. Moreover, the GPE score measured at T1 was 2.20 considering patients' perspective and 2.40 considering physical therapists' perspective.

**Table 3:** Differences in outcome measures from baseline (T0) to the end of 4-week rehabilitation treatment (T1) and the follow-up assessment at 3 months from the baseline (T2).

	**T0**	**T1**	**T0-T1 *P*-value**	**T2**	**T0-T2 *P*-value**
BFI	5.4 ± 1.6	4.2 ± 1.7	0.004	4.4 ± 1.6	0.004
FFM (kg)	43.2 ± 6.4	44.4 ± 6.2	0.231	45.5 ± 6.6	<0.001
FM (kg)	24.0 ± 10.6	22.9 ± 10.2	0.297	21.7 ± 10.0	<0.001
HGS (kg)	20.1 ± 5.8	22.5 ± 5.2	<0.001	21.7 ± 6.0	0.012
SPPB	9.3 ± 2.0	11.3 ± 1.2	<0.001	11.7 ± 0.5	<0.001
10 MWT (m/s)	1.5 ± 0.3	1.8 ± 0.3	<0.001	1.9 ± 0.3	<0.001
6 MWT (m)	464.5 ± 62.9	554.1 ± 71.6	<0.001	567.1 ± 82.7	<0.001
**EORTC QLQ-C30**					
Functional score	69.2 ± 14.9	76.9 ± 15.7	<0.001	75.0 ± 17.1	0.005
Symptoms score	29.2 ± 14.9	21.2 ± 16.0	<0.001	21.9 ± 18.5	<0.001
Global Health score	40.7 ± 12.5	67.6 ± 14.8	<0.001	65.2 ± 20.0	<0.001

*BFI, Brief Fatigue Inventory; FFM, fat-free mass; FM, fat mass; HGS, hand-grip strength test; SPPB, Short Physical Performance Battery; 10 MWT, 10-min walking test; 6 MWT, 6-min walking test; EORTC QLQ-C30, European Organization for Research and Treatment of Cancer Quality of Life Questionnaire*.

## Discussion

Several exercise programs have been proposed to reduce BCF (17, 32–36). However, the choice of the most appropriate intervention to offer remains troubled in real-life clinical practice. Here, we subjected an exploratory cohort of breast cancer survivors suffering from BCF to a physical exercise rehabilitative protocol consisting of 10 min of warm-up, 40 min of aerobic exercise and strength training, and 10 min of cool-down, twice a week for 4 weeks. Taken together, a significant decrease of BCF was observed at the end of the program and was maintained at the follow-up visits.

To date, there is still little evidence about the multifactorial mechanisms underpinning BCF pathogenesis. Historically, the loss of muscle mass, metabolism disorders and ATP production impairment have been viewed as founder events (37). In our study, we noticed a significant improvement in the FFM and a significant reduction of the FM at a 2-months follow-up but not at T1, suggesting that muscle mass modifications need more time to manifest compared to the relatively fast improvement of all the functional outcomes assessed. These results highlight that rehabilitative physical exercise counters the main mechanisms underpinning BCF and might be considered as an effective and reliable treatment option. This notion, however, should be considered in the context of the small sample size investigated in the present work.

A recent randomized controlled trial investigated the effects of a specific training program to modulate systemic inflammation (38). After this intervention, serum levels of TNF-α, IL-6, and IL-10 were significantly lower in the intervention group, confirming the anti-inflammatory properties of physical exercise previously demonstrated in several pathological conditions (39–42) and suggesting a possible mechanism through which it intervenes in countering BCF clinical manifestations. Of note, given the lack of adverse events in our study group, we confirm the excellent safety profile of this physical exercise intervention. This approach proved to be feasible, considering the high treatment adherence (i.e., no dropouts) and the high GPE scores obtained by both patients and physical therapists.

The skeletal muscle system has been recently hypothesized to have a key role in fatigue pathogenesis (43, 44). Furthermore, there are multiple examples in literature on the direct mitochondria damage, inducing a dysfunction characterized by an increased intracellular oxidative stress and low energy supply (8, 45–48). Noteworthy, exercise training could remodel the mitochondrial network, influencing the mitochondria intrinsic plasticity through different mechanisms and modulating their shape in response to fission and fusion events (45, 47, 49–51). Furthermore, it has been recently proved that physical exercise is able to improve mitochondrial function and dynamics in fragile patients (51). Similarly, an endurance exercise protocol could have a key role in the prevention of muscle wasting by stimulating mitochondrial dynamics. Taken together, all these findings, coupled with our preliminary observations, could suggest that exercise therapy might have a crucial impact not only in the clinical and therapeutic management of BCF, but also interfering directly in its pathogenesis.

This study has several limitations. First, the relatively small sample size of women with BCF included in the study could have limited the clinical impact of our conclusions. It should be noted, however, that our pilot prospective study provides for the first time in literature evidence on the possible clinical application of a specific physical exercise rehabilitative treatment in this setting. Further prospective studies embracing larger cohorts of patients are warranted to define the implications of our observations. Second, due to the study design, we did not collect any data on bone mineral density, falls, and fracture rate. Indeed, a high prevalence (80.6%) of patients treated with aromatase inhibitor therapy, a well-known risk factor for osteoporosis (52), has been recruited in the present study. Considering the beneficial effects of physical exercise on bone mineral density in premenopausal and postmenopausal women (53), the improvement of all functional parameters that we observed after a 1-month protocol might constitute the basis for a possible role in contrasting osteoporosis, reducing the risk of falling and consequently the risk of fragility fractures. On the other hand, given that these women are at high risk of osteoporosis, it is mandatory to underline the role of an adequate therapeutic exercise to prevent fractures and all the disabling consequences. Third, the lack of a control group limits the translational relevance of our hypothesis. However, this study should be considered a proof-of-principle that rehabilitation interventions can be safety and effectively performed in breast cancer survivors. Lastly, we did not provide any data on long-term outcomes because all of the patients enrolled in this prospective study are still followed up by our multidisciplinary team.

Despite these limitations, we provide preliminary and previously unavailable evidence on the feasibility, reliability, and safety of a 1-month specific physical exercise rehabilitative protocol in reducing BCF, improving muscle mass, muscular-skeletal function, and health-related quality of life in breast cancer survivors. Our results advocate the need to define tailored physical exercise interventions that could be performed in common clinical practice as a first-line rehabilitative treatment to reduce BCF.

## Data Availability Statement

The raw data supporting the conclusions of this article will be made available by the authors, without undue reservation.

## Ethics Statement

The studies involving human participants were reviewed and approved by Comitato Etico Interaziendale Novara. The patients/participants provided their written informed consent to participate in this study.

## Author Contributions

MI and CCi: study concept and design. MI, CCi, and NF: supervision. MI and AS: manuscript writing (first draft). AS, LL, KV, FG, and AG: bibliography. MI: iconography. KV, ES, CCr, and NF: critical revision. All authors contributed to the article and approved the submitted version.

## Conflict of Interest

The authors declare that the research was conducted in the absence of any commercial or financial relationships that could be construed as a potential conflict of interest.
